# Effect of sleep loss on pain—New conceptual and mechanistic avenues

**DOI:** 10.3389/fnins.2022.1009902

**Published:** 2022-12-20

**Authors:** Kamila Kourbanova, Chloe Alexandre, Alban Latremoliere

**Affiliations:** ^1^Department of Neurosurgery, Neurosurgery Pain Research Institute, Johns Hopkins School of Medicine, Baltimore, MD, United States; ^2^Department of Neuroscience, Johns Hopkins School of Medicine, Baltimore, MD, United States

**Keywords:** sleep deprivation, pain, nucleus accumbens, DNIC, NREMS, REMS, forced awakening, SWS

## Abstract

**Introduction:**

Sleep disturbances increase pain sensitivity in clinical and preclinical settings, but the precise mechanisms are unknown. This represents a major public health issue because of the growing sleep deficiency epidemic fueled by modern lifestyle. To understand the neural pathways at the intersection between sleep and pain processes, it is critical to determine the precise nature of the sleep disruptions that increase pain and the specific component of the pain response that is targeted.

**Methods:**

We performed a review of the literature about sleep disturbances and pain sensitivity in humans and rodents by taking into consideration the targeted sleep stage (REMS, non–NREMS, or both), the amount of sleep lost, and the different types of sleep disruptions (partial or total sleep loss, duration, sleep fragmentation or interruptions), and how these differences might affect distinct components of the pain response.

**Results:**

We find that the effects of sleep disturbances on pain are highly conserved among species. The major driver for pain hypersensitivity appears to be the total amount of sleep lost, while REMS loss by itself does not seem to have a direct effect on pain sensitivity. Sleep loss caused by extended wakefulness preferentially increases pain perception, whereas interrupted and limited sleep strongly dysregulates descending controls such as DNIC, especially in women.

**Discussion:**

We discuss the possible mechanisms involved, including an increase in inflammatory processes, a loss of nociceptive inhibitory pathways, and a defect in the cognitive processing of noxious input.

## Introduction

When individuals do not get sufficient sleep or have their sleep curtailed, they fail to maintain normal levels of alertness, which translates into attentional lapses and poor cognitive performances (Van Dongen et al., 2003; Goel et al., 2009; McHill et al., 2018). Insufficient sleep also negatively impacts metabolism (Spiegel et al., 1999; Tasali et al., 2008), immune and cardiovascular functions (Faraut et al., 2012; Tobaldini et al., 2014; Kohansieh and Makaryus, 2015; Irwin et al., 2016), emotional regulation (Walker, 2009; Simon et al., 2015), and pain (Smith and Haythornthwaite, 2004; National Sleep Foundation, 2015). The overall enhancement in pain sensitivity caused by various sleep disturbances has been widely reported both at the clinical and preclinical levels with consistent effects across species (Lautenbacher et al., 2006; Finan et al., 2013; Schrimpf et al., 2015; Alexandre et al., 2020). In sharp contrast, sleep disturbances do not increase sensitivity to innocuous stimuli of various sensory modalities: thermal discrimination, responsivity to light mechanical stimuli, auditory and visual acuities are not changed by sleep loss (Kundermann et al., 2004; Franzen et al., 2009; Scherer et al., 2013; Alexandre et al., 2017). Altogether, these results indicate that inadequate sleep specifically enhances pain sensitivity, but the precise mechanisms responsible remain largely unknown.

**Figure 1:**
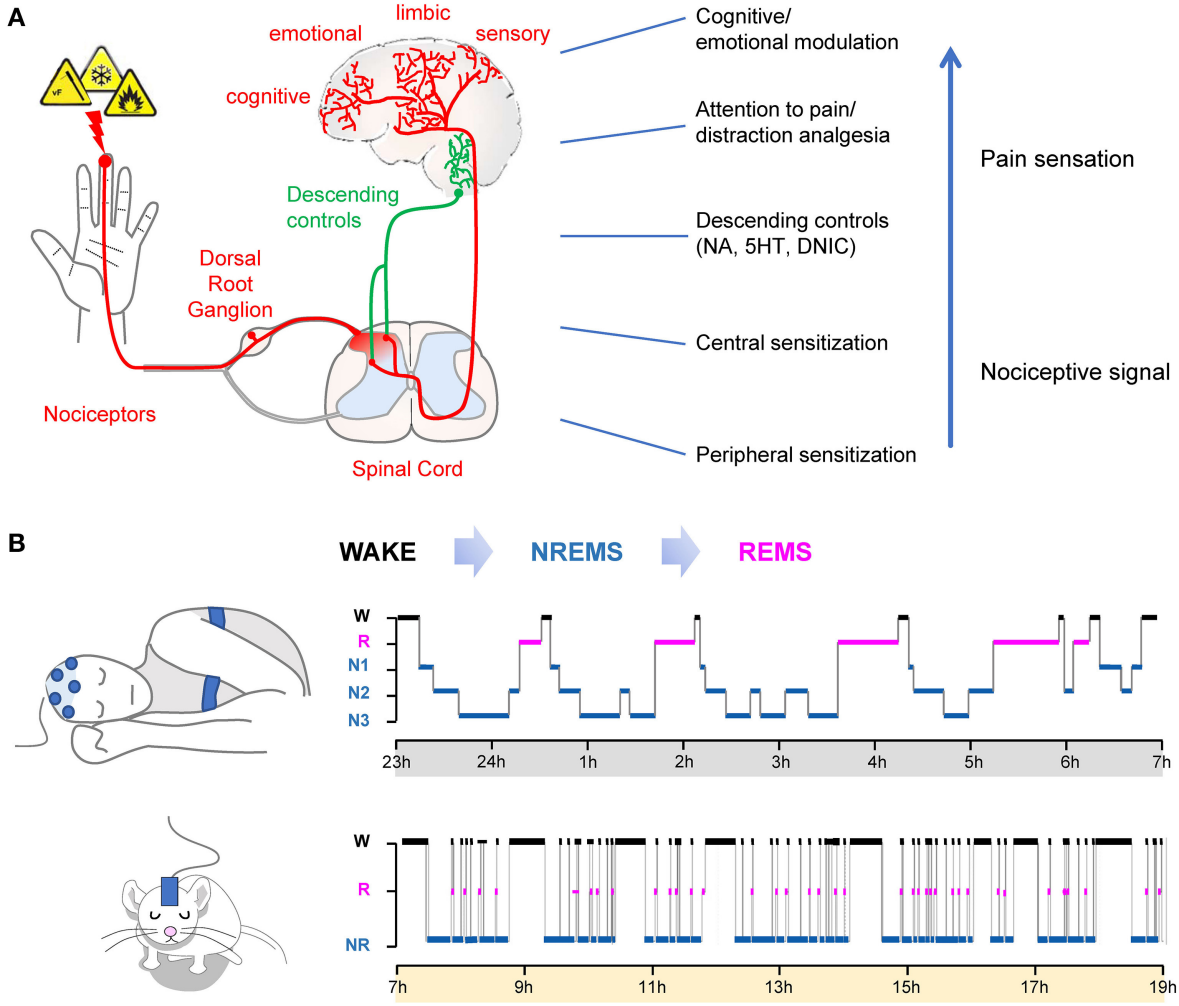
**(A)** Schematic representation of nociceptive and pain pathways. Nociception consists of the detection of noxious stimuli, spinal reflexive withdrawal and the transmission of the signal into the brain, where the pain sensation is generated. The pain sensation comprises sensory-discriminative, emotional and cognitive components. Right: Example of major pain/nociception modulatory processes. **(B)** Sleep-wake cycles in humans (top) and rodents (bottom) as analyzed with polysomnography. In normal conditions, NREMS always precedes REMS. Humans display consolidated sleep during the night period, while rodents have polyphasic sleep, most prominent during the light period (subjective sleep phase).

Pain is a complex neural process that involve detecting a potentially harmful stimulus *via* specialized peripheral sensory neurons called nociceptors, integrating the information at the spinal cord level before sending it to the brain where a pain sensation is generated to incorporate sensory-discriminative, emotional, limbic and even cognitive components (Figure 1A). Virtually all the components of the pain response can be modulated along the nociceptive and pain pathways to either enhance or depress the pain signal (Figure 1A). The ultimate pain sensation generated is the result of the integration of all these pain components, which contributes to the multifactorial and individualized features of pain. Therefore, an overall increase in pain sensitivity can be obtained by various mechanisms, raising the possibility that different sleep disturbances alter pain processing at distinct levels of the nociceptive and pain pathways, and thus could have cumulative effects.

Sleep is also a complex and dynamic neural process that comprises of two major and distinct states, rapid eye movement (REM) sleep and non–rapid eye movement (NREM) sleep. They alternate periodically throughout the sleep cycle and can be distinguished based on their electroencephalogram (EEG) and electromyogram (EMG) features (Aserinsky and Kleitman, 1953). In normal conditions, sleep architecture has a predictable macro-organization with NREMS always preceding REMS (Figure 1B). In humans, three stages of NREMS have been characterized based on their EEG features and to reflect transition into “deeper” sleep (N1, N2, and N3 which is also designated as slow wave sleep SWS). NREMS intensity can be assessed by quantifying the EEG power between 0.5 and 4 Hz, also known as slow-wave activity (SWA) (Borbely, 1982; Borbely and Achermann, 1999). REMS is characterized by an “activated” EEG (high frequency, low amplitude waves), no postural muscular activity on the EMG (atonia), and ocular saccades (that can be quantified with electrooculogram) in both humans and rodents. The distribution and amount of NREMS and REMS are mostly determined by two principal and interacting systems: the endogenous circadian clock and a homeostatic component that regulates the need and intensity of sleep according to the sleep-wake history (i.e., the time previously spent asleep or awake) (Borbely, 1982). Due to these dynamic interrelations between sleep-wake stages, many experimental sleep disruptions are poised to have broader effects on other sleep parameters than originally anticipated, notably NREMS and REMS amount. As a result, the exact effects of various sleep disturbances, especially during chronic settings, on pain processing are not clear.

To understand the neural pathways at the intersection between sleep and pain processes, it is then critical to define precisely which sleep stages (REMS, non–NREMS, or both) and what type of the sleep disruptions (sleep loss, sleep fragmentation or sleep interruptions) affect the pain sensation.

Here, we performed a review of the literature about sleep disturbances and pain sensitivity in humans and rodents by taking into consideration the amount of sleep lost. We find that NREMS loss could be the major driver for pain hypersensitivity, and that the temporal nature of the sleep disruptions could affect different components of the pain response. We then discuss possible neural mechanisms responsible and cellular pathways involved.

## Part 1: Total (NREMS+REMS) sleep loss on pain

**Figure 2:**
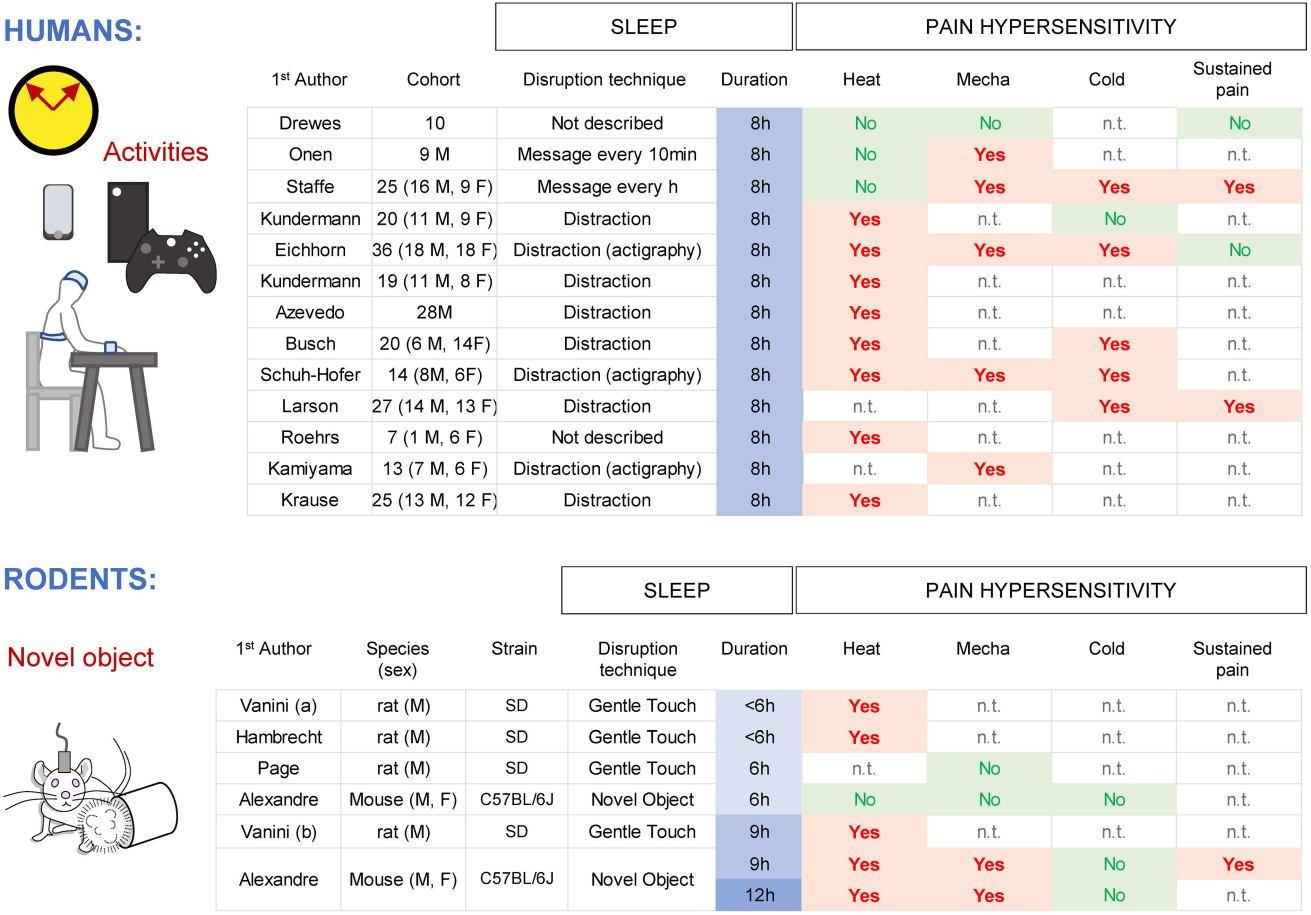
Effects of total sleep deprivation in humans and rodents for different pain modalities (heat, mechanical, cold) and sustained pain. M indicates men and F indicates women. n.t., not tested.

In humans, a single night of total sleep deprivation caused a decrease in pain thresholds from a heat source (8 studies out of 11), mechanical stimulus (both punctate and pressure; 5 studies out of 6) and cold (5 studies out of 6) when tested immediately at the end of the sleep deprivation (Figure 2). Furthermore, the pain response to a sustained noxious stimulus (ischemia pain, cold pressor pain) was heightened in half of the studies, suggesting a less robust or a ceiling effect of the pain response (Drewes et al., 1997; Onen et al., 2001; Larson and Carter, 2016; Eichhorn et al., 2018; Staffe et al., 2019). Spontaneous pain complaints (general body pain, low back pain, or headache) and migraine crises are more often reported after one night of total sleep deprivation (Busch et al., 2012; Houle et al., 2012; Palma et al., 2013). Both women and men show an hyperalgesic effect of sleep loss, with a stronger trend for women (Eichhorn et al., 2018; Staffe et al., 2019). One night of total sleep deprivation increased temporal summation of pain ratings upon repeated noxious pressure stimulations (Staffe et al., 2019), suggesting a possible spinal nociceptive facilitation (Bosma et al., 2015), although amplification within the brainstem, thalamus, or cortical areas might also contribute. In contrast, detection of innocuous stimuli (gentle touch, warmth) was not affected by sleep loss (Kundermann et al., 2004, 2008; Lautenbacher et al., 2006; Schuh-Hofer et al., 2013). Other sensory systems such as visual performance and acoustic startle/reactivity, showed a decrease in responsiveness after sleep loss (Franzen et al., 2009; Jung et al., 2011; Liberalesso et al., 2012; Scherer et al., 2013).

In rodents, acute total sleep deprivation is usually performed by variations of the gentle handling paradigm (Tobler et al., 1997; Page et al., 2014; Vanini et al., 2014; Vanini, 2016; Alexandre et al., 2017) with animals tested at the end of the session. Protocols providing novel objects without touching the animals are minimally stressful, allowing for an extension of wakefulness without forced locomotor activity. Nine and twelve hours of sleep deprivation caused an increased sensitivity for noxious heat (hotplate set at 52 C, radiant heat with Hargreaves), pressure (von Frey filaments) and sustained pain (capsaicin), with an overall trend toward higher responses in females (Page et al., 2014; Vanini et al., 2014; Vanini, 2016; Alexandre et al., 2017; Hambrecht-Wiedbusch et al., 2017). Responses to noxious cold (~4 C; acetone test) were not changed by sleep loss, in contrast to human data, which might be due to technical differences to generate cold (Harvey et al., 2010; Alexandre et al., 2020). Responses to somatic innocuous stimuli (gentle touch with a brush, warmth (20–37 C) detection though a thermal gradient) were not changed, even after 12 h of sleep deprivation (Alexandre et al., 2017). Startle responses following loud acoustic stimuli were decreased after 9 h of sleep loss (Alexandre et al., 2017), similar to humans and presumably reflecting fatigue. Overall, these results indicate that the enhancement of pain responses by total sleep loss is consistent and well preserved in both rodents and humans.

## Part 2: State-specific sleep deprivation in humans

In humans, SWS (NREMS N3) can be interrupted by monitoring the EEG/EMG signals and applying a short auditory stimulus (80–110 dB) based on the EEG/EMG features observed in real time [for N3 = detection of slow-waves (0.5–3.5 Hz)]. This protocol does not cause a total loss of NREMS or REMS, but it increases the time spent in N1 and N2 (Arima et al., 2001). Three consecutive nights of SWS deprivation did not affect pressure pain responses applied to jaw muscles (Arima et al., 2001), while pain sensitivity to pressure at tender points (commonly used to diagnose fibromyalgia) was reported increased in some studies (Moldofsky et al., 1975; Moldofsky and Scarisbrick, 1976; Lentz et al., 1999) but not in others (Older et al., 1998). All these studies had a relatively low N (ranging from 6 to 19) which could explain the variability and possibly insufficient statistical power. No major differences on pain sensitivity were found between men and women. There is currently no specific SWS-deprivation protocol in rodents.

**Figure 3:**
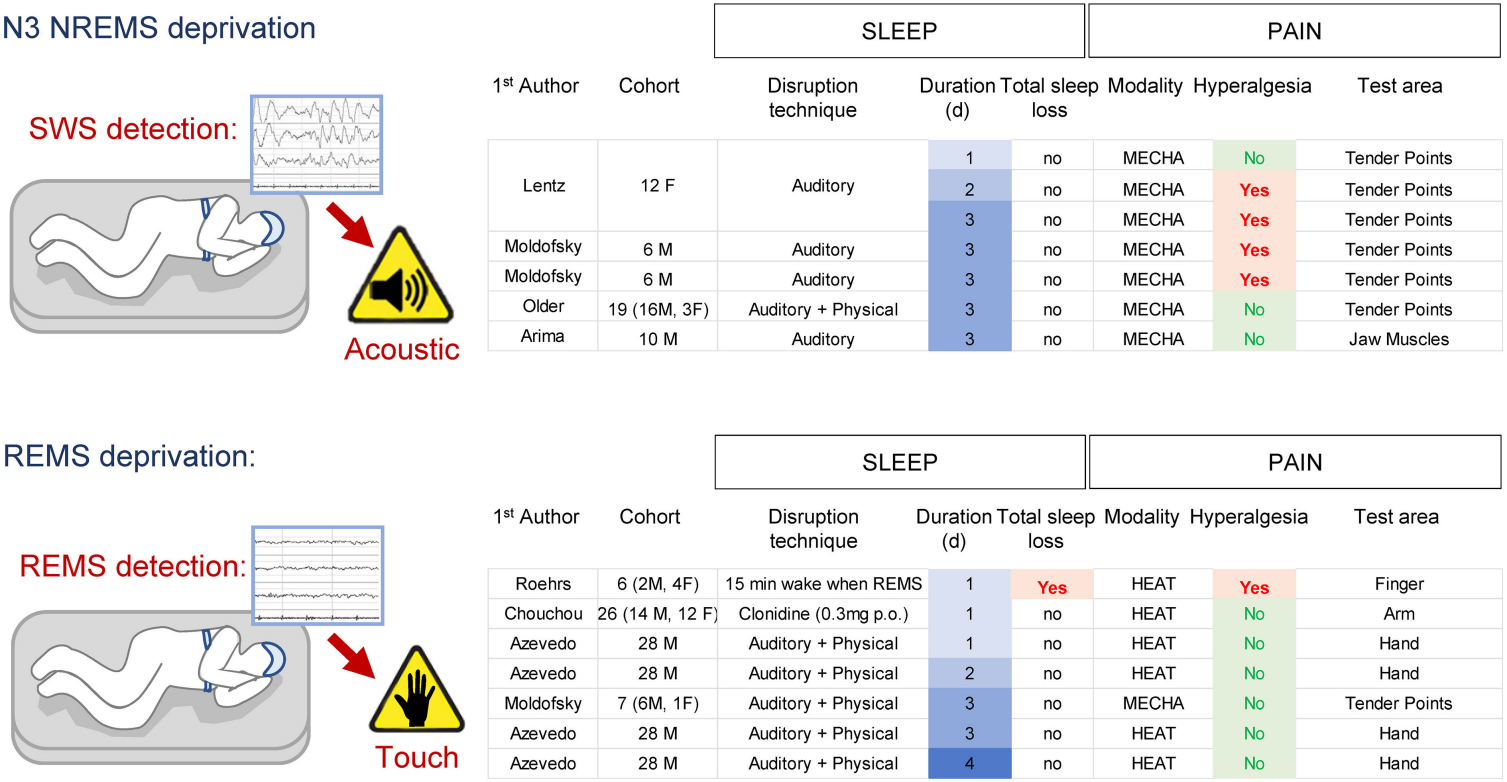
Effects of SWS- and REMS-specific deprivation on pain in humans. Note the associated total sleep loss occurring in one REMS deprivation protocol associated with pain hypersensitivity.

REMS can be interrupted in humans by a frank awakening (e.g., touching or moving subject) based on EEG/EOG/EMG features observed in real-time. Quantifying the total amount of sleep under these protocols indicates that some REMS deprivation protocols might also cause a significant loss of NREMS (~30%; Roehrs et al., 2006), while others do not (Moldofsky and Scarisbrick, 1976; Azevedo et al., 2011). Interestingly, REMS deprivation protocols that are not associated with total sleep loss do not increase pain even after 4 consecutive days (Azevedo et al., 2011), while a protocol that reduced total sleep amount increased heat pain sensitivity after a single night (Roehrs et al., 2006) (Figure 3). REMS deprivation by administration of clonidine did not change heat pain sensitivity (nor total sleep amount) in either men or women (Chouchou et al., 2015).

## State-specific sleep deprivation in rodents

**Figure 4:**
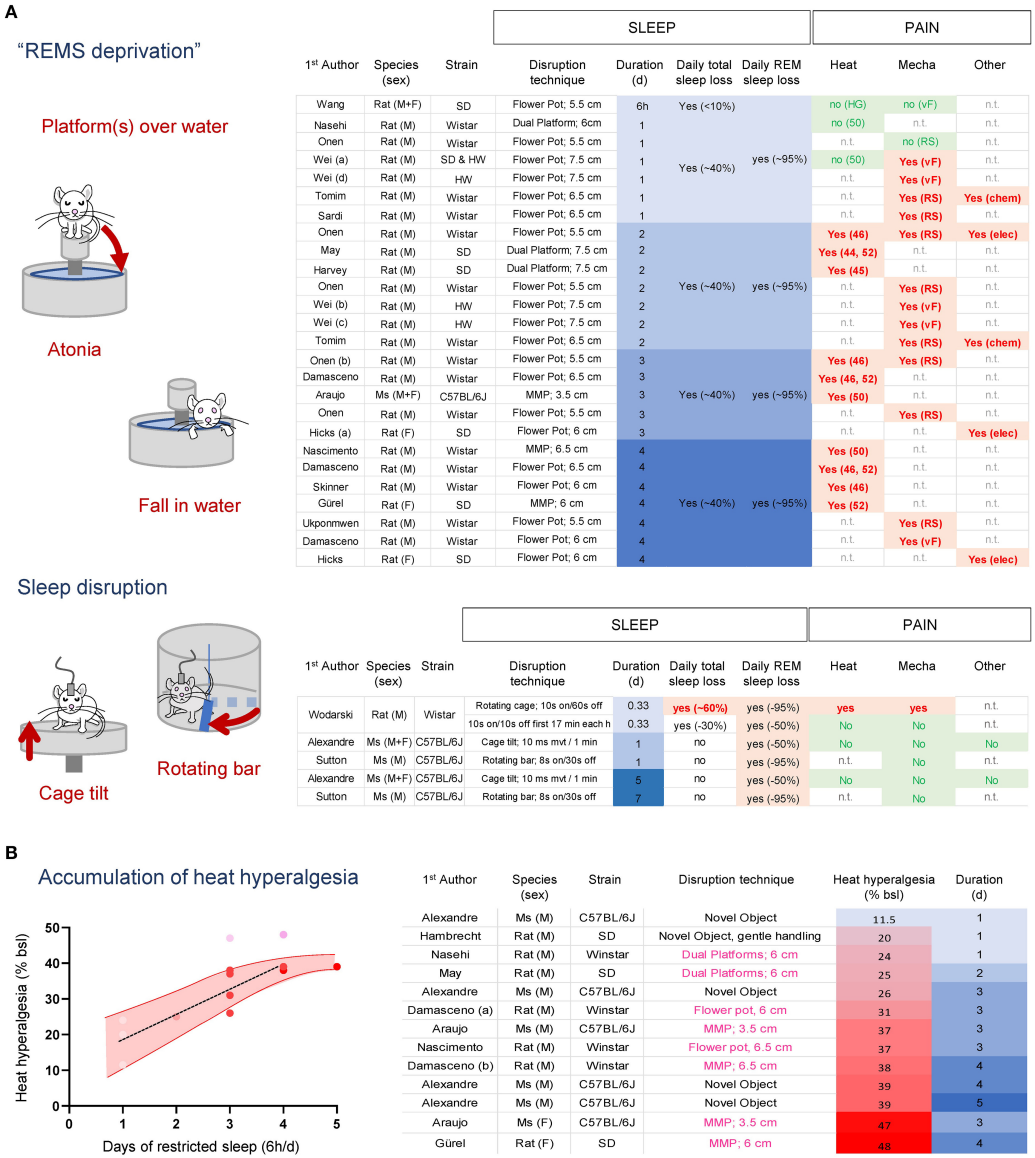
**(A)** Effects of “platform over water” methods and sleep disruption protocols on sleep and heat and mechanical pain in rodents. REMS loss alone is not associated with the development of hyperalgesia, while protocols that cause total sleep loss increase pain sensitivity. **(B)** Heat hyperalgesia development accumulates over time independent of the species or sleep deprivation protocols used. An estimated 6 h of total sleep lost per day causes a relatively linear increase in heat pain that plateaus after 4 days. Heat hyperalgesia was estimated as a % of baseline from the studies included. Studies with males are shown in red dots; studies with females are shown in pink dots. SD, Sprague Dawleys; HW, Hanover Winstar; HG, Hargreaves test; (46–52), hotplate set at 46–52 C; RS, Randall & Selitto pressure test; vF, von Frey assay; Chem, Formalin 1%; Elec, electric current.

In rodents, the majority of REMS sleep deprivation has been performed using variations of the “platform-over-water” method (Mendelson et al., 1974) that take advantage of the muscle atonia occurring during REMS (Jouvet et al., 1964). Rats or mice are placed on top of a small platform surrounded by water in which they fall when they enter REMS and lose postural tone, causing them to wake up (Figure 4A). A modification of this paradigm was introduced to circumvent isolation and movement restriction by allowing a socially stable group of animals to be sleep-deprived together in a large tank containing multiple small platforms (Coenen and Van Luijtelaar, 1985). While 6 h of this paradigm does not change heat and mechanical pain sensitivity and 24 h has limited effects (Onen et al., 2000; Wei et al., 2007; Wang et al., 2015; Tomim et al., 2016; Nasehi et al., 2018; Sardi et al., 2018b), two or more consecutive days are systematically associated with an increase in pain responses for mild (44–46 C) (Onen et al., 2000; May et al., 2005; Damasceno et al., 2009; Harvey et al., 2010; Skinner et al., 2011) and intense (52 C) (Nascimento et al., 2007; Damasceno et al., 2009; Araujo et al., 2011; Gurel et al., 2014) heat, pressure (Randall and Selitto) (Ukponmwan et al., 1984; Onen et al., 2000; Tomim et al., 2016) and punctate (von Frey filaments) (Wei et al., 2008, 2010; Damasceno et al., 2009) mechanical stimuli, and chemical pain (Figure 4A) (Hicks et al., 1979; Onen et al., 2000; Tomim et al., 2016). The development of heat hyperalgesia is stronger in females (Araujo et al., 2011). However, “platform-over-water” protocols significantly reduce NREMS by ~30–50%, nearly ~4–6 h sleep loss per day (Grahnstedt and Ursin, 1985; Machado et al., 2004), making it difficult to distinguish the relative contribution of REMS or NREMS loss to the development of hyperalgesia. Interestingly, the overall onset of hyperalgesia during “platform-over-water” protocols ranges between 24 and 48 h, (corresponding to ~5–10 h total sleep loss), which is very similar to that of an acute total sleep deprivation (between 6 and 9 h) (Alexandre et al., 2017).

Sleep disruption protocols aiming at fragmenting NREMS can also indirectly prevent the transition from NREMS to REMS, thereby reducing REMS amount without significantly altering NREMS amount in most cases (Figure 4B). These protocols usually involve circular cages equipped with a rotating sweeping bar or cage floor programmed to rotate/move at specific intervals and durations (Figure 4B) (Ringgold et al., 2013; Sutton and Opp, 2014; Alexandre et al., 2017). Under these protocols, loss of REMS (ranging from 50 to 95%) without concomitant loss of total sleep did not alter mechanical or heat pain after one or up to 5 consecutive days in males or females (Sutton and Opp, 2014; Wodarski et al., 2015; Alexandre et al., 2017). In one study (Wodarski et al., 2015), 8 h of sleep fragmentation that caused a minor total sleep loss (~30%; 2 h 40 min) did not increase heat and mechanical pain responses, while a sleep fragmentation protocol that also caused ~60% total sleep loss (~5 h 30 min) increased pain responses. Such estimation of the amount of total sleep loss required to increase pain sensitivity is aligned with total sleep deprivation studies (Page et al., 2014; Alexandre et al., 2017).

Overall, these results suggest that traditional “REM sleep-specific” deprivation protocols relying on platforms-over-the-water techniques cause a significant total sleep loss over time, which might be the main driver for pain hypersensitivity, rather than the specific loss of REMS. Indeed, when ranking all studies that used the same pain assay (latency to withdraw on a hotplate set a 50–52 C; to mitigate technical variability) by pain intensity, we found that the main factor associated with the gradual increase in heat pain was the overall amount of sleep loss, irrespective of the sleep disruption protocol or species used (Figure 4B). Furthermore, these compiled studies suggest that when subjected to ~6 h total sleep loss per day, animals develop heat hyperalgesia with a relatively linear increase of ~7% per day and reach a plateau after 4 d (Figure 4B). There were not enough studies carried out with females to perform this analysis. The overall pain profile from acute and chronic sleep loss experiments suggests females develop stronger hyperalgesia and might reach a plateau sooner compared to males. Altogether, these results fit with the hypothesis that the amount of total sleep loss, and not REMS or sleep continuity, is the major factor associated with the increased pain sensation for heat and mechanical stimuli.

## Part 3: Dynamics of sleep loss on pain

**Figure 5:**
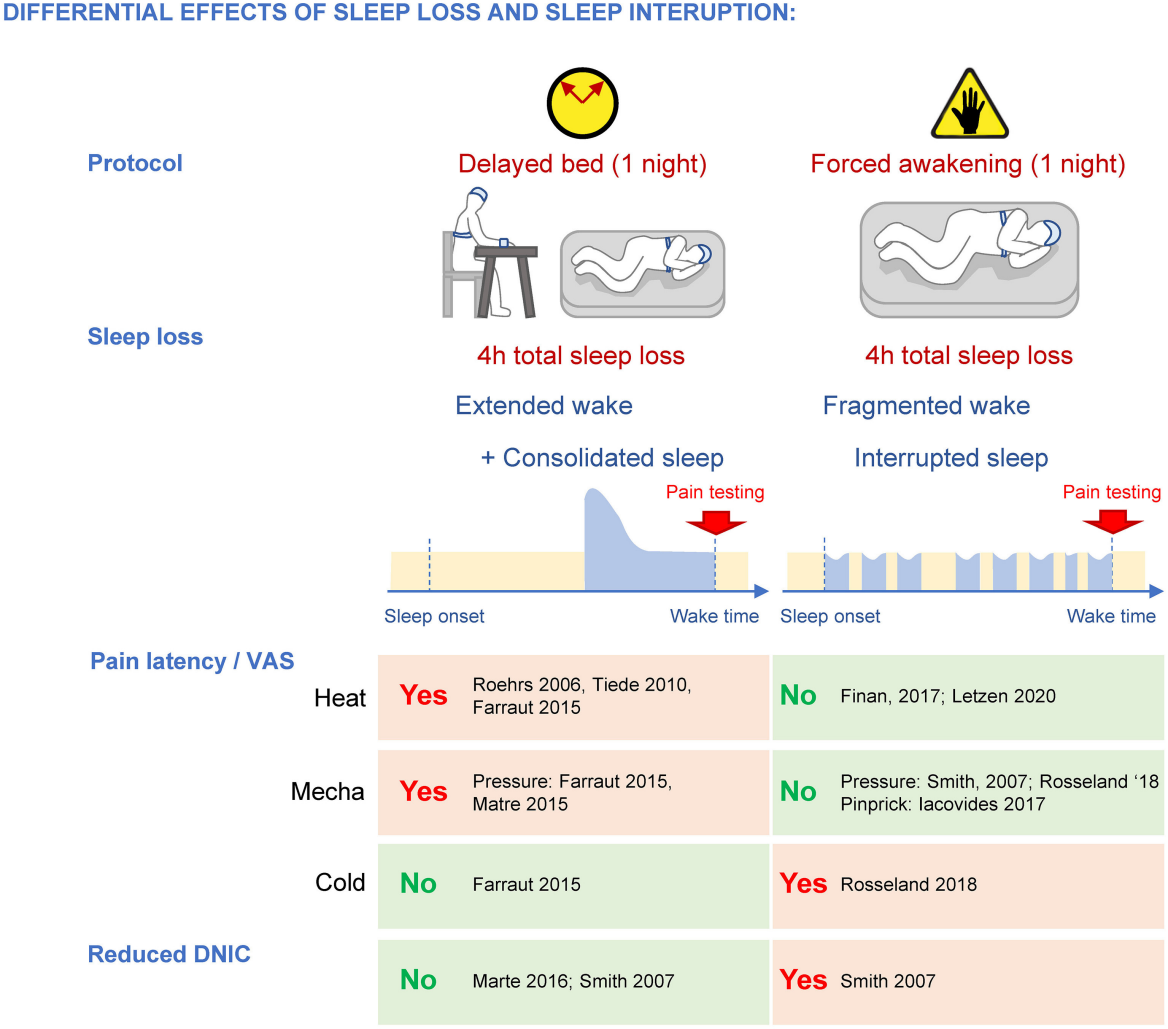
Differential effects of sleep loss caused by extended wake and fragmented wake/interrupted sleep on pain sensitivity and DNIC. While both protocols cause a loss of total sleep of ~4 h, extended wake promotes heat and mechanical pain hypersensitivity and forced awakenings disrupt DNIC specifically.

While the amount of total sleep loss appears to be a major contributor to the increased pain response, the dynamics of the sleep loss could target different components of pain processing. This can be evidenced when comparing two models of sleep disruption in humans achieving the same sleep deficit after a single night: delayed bedtime and forced awakening (Smith et al., 2007). In the delayed bedtime protocol, subjects stay awake for 4 h past their usual bedtime, while under the forced awakening protocol, subjects are awakened for 20 min every hour, and for a full hour at a random time during the night. Both protocols reduce total sleep amount by ~45–50% (~4 h). While the delayed bedtime protocol initially extends wakefulness then allows for a consolidated sleep (i.e., with increased SWS during the remaining sleep opportunity), the forced awakenings protocol interrupts sleep with transient, short (20 min to 1 h) wake periods (causing more N1 at the expense of SWS) (Figure 5). These different dynamics of sleep loss are associated with preferential changes in pain responses, possibly underlying specific mechanisms. In both men and women, a single 4-h delayed bedtime is sufficient to increase heat and mechanical pain sensitivity when subjects are tested at wake up (Roehrs et al., 2006; Tiede et al., 2010; Faraut et al., 2015; Matre et al., 2015), similar to the effects of a night of total sleep deprivation, while a single night of forced awakenings does not affect these modalities (Smith et al., 2007; Finan et al., 2017; Iacovides et al., 2017; Letzen et al., 2020). Both delayed bed onset and total sleep deprivation are associated with an extended wake, suggesting that heat and mechanical pain might be caused by a common mechanism sensitive to excessive wakefulness, i.e., a maximum period beyond which normal pain perception and neurobehavioral functioning cannot be maintained (Van Dongen et al., 2003). In support of this, administration of caffeine and modafinil, two drugs that can restore alertness, normalize heat and mechanical pain sensitivity (Vanini, 2016; Alexandre et al., 2017), without removing the sleep debt. Both sleepiness and pain hypersensitivity accumulate during experimental sleep deprivation (Alexandre et al., 2017), and sleepiness ratings in healthy individuals correlate with their pain complaints (Smith et al., 2007). Chemogenetic activation of GABAergic neurons in the anterior cingulate cortex in mice is sufficient to concomitantly increase delta power (which could indicate increased sleep pressure) and mechanical pain (Li et al., 2020). These results suggest that the neural circuits driving sleepiness could also be responsible for the heightened heat and mechanical pain sensitivity after an acute sleep loss.

Another major difference in pain processing between reduced yet consolidated sleep and fragmented sleep concerns diffuse noxious inhibitory controls (DNIC), a type of descending controls that can suppress the nociceptive signal at the spinal cord level. DNIC are triggered when two noxious stimulations occur simultaneously in different dermatomes, and they can be assessed in humans by using “conditioned pain modulation (CPM)” protocols (Yarnitsky, 2010; Yarnitsky et al., 2015). One night of forced awakenings strongly disrupts CPM in women (Smith et al., 2007), whereas a single 4-h delayed bedtime does not (Matre et al., 2016; 14 women, 8 men; Smith et al., 2007; women only; Matre et al., 2017; night shifts: 41 women, 12 men) (Figure 5), indicating that a mild sleep loss with disrupted continuity specifically targets these descending controls. Clinical studies support this assertion as DNICs are strongly altered in fibromyalgia patients who also suffer from mild total sleep loss and sleep disruption by awakenings (lasting more than 3 min; Lautenbacher and Rollman, 1997; Diaz-Piedra et al., 2015; Bjurstrom and Irwin, 2016), as well as patients with temporomandibular joint pain where DNICs were most altered in patients with poor sleep quality (Edwards et al., 2009). Interestingly, experimental sleep disturbances only disrupt DNIC in women (Eichhorn et al., 2018), which could contribute to the strong incidence of fibromyalgia and temporomandibular joint disorder pain in women.

DNICs are triggered specifically by the activation of lamina V spinal neurons (Villanueva and Le Bars, 1995) that project to the subnucleus reticularis dorsalis (SRD, also known as dorsal reticular nucleus DRt, and called medullary reticular nucleus in mice; Villanueva and Le Bars, 1995; Le Bars, 2002). Some SRD neurons project directly back into the deep dorsal horn lamina (V-VII; Villanueva et al., 1995) while others activate several medullar pathways (Bouhassira et al., 1992b) that will also eventually project back to the spinal cord to inhibit projecting neurons (Leite-Almeida et al., 2006). It is important to note that DNICs are physiologically distinct from “traditional” descending inhibitory controls that originate from the rostroventral medulla (RVM), notably the locus coeruleus (LC) and the median raphe nucleus (MRN) (Basbaum and Fields, 1978, 1984; Bouhassira et al., 1992a; Le Bars et al., 1992; Kucharczyk et al., 2022), and the effects of forced awakenings on those are not known. Nonetheless, because delayed bed onset does not disrupt DNICs, this raises the possibility that a mild sleep loss coupled with sleep interruptions caused by the forced awakening protocol might affect more strongly lamina V pain pathways.

In contrast to heat and mechanical pain, cold pain responses are not changed by delayed bed protocol (Faraut et al., 2015), but they are increased after forced awakenings (Rosseland et al., 2018) (Figure 5).

## Part 4: Chronic sleep disruptions and recovery sleep

The similar increase in acute pain responses to heat and mechanical stimuli observed after a single night of total sleep deprivation or at wake time after a delayed bedtime indicates that a short sleep opportunity is not sufficient to restore normal pain sensitivity. Indeed, the increase in heat and mechanical pain caused by sleep loss persists in humans (Roehrs et al., 2006; Tiede et al., 2010; Faraut et al., 2015; Matre et al., 2015, 2016; Odegard et al., 2015; Staffe et al., 2019) and even aggravates in rodents (Page et al., 2014; Alexandre et al., 2017; Sardi et al., 2018a). Cumulative deficits after sleep loss have also been reported for diverse biological processes, such as cognitive performance (Van Dongen et al., 2003; Banks et al., 2010), energetic metabolism (Spiegel et al., 1999; Van Cauter et al., 2008; Everson and Szabo, 2011), and immune system functions (Bryant et al., 2004). This is particularly relevant, because chronic insufficient sleep is becoming more frequent in the general population (up to 30%) and can be caused by poor sleep hygiene (TV, screens, etc.), work conditions (schedules/shiftwork, multiple jobs) or diseases (e.g., obesity-related sleep disorders; Holingue et al., 2018; Freire et al., 2022).

**Figure 6:**
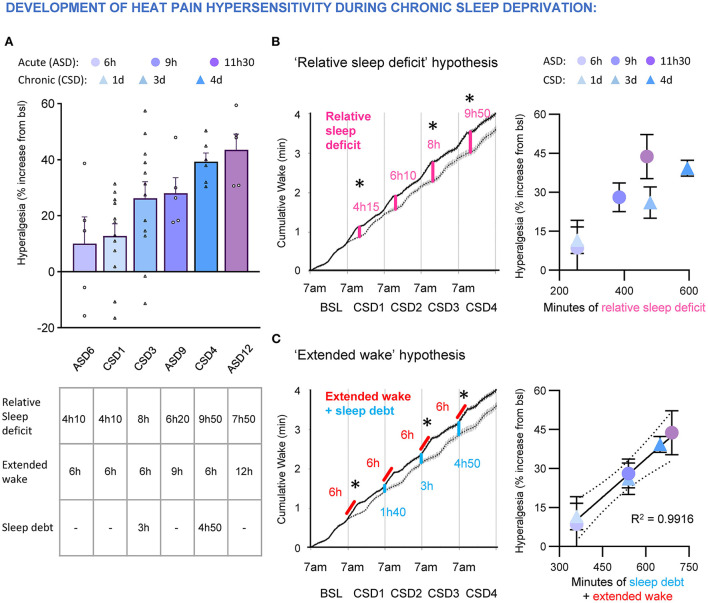
**(A)** Top, development of heat hyperalgesia after acute sleep deprivation 6, 9, and 12 h (ASD6, 9, 12) and chronic sleep deprivation (6 h of sleep deprivation per day; CSD1, 3, 4). Data are expressed as % of baseline and ranked by intensity of hyperalgesia. Each dot represents an animal. Bottom, table indicating the amount of relative sleep deficit (compared to undisturbed sleep), extended wake and sleep debt at the beginning of sleep deprivation. Data are presented as cumulative hours of wake from 7 a.m. **(B)** “Relative sleep deficit” model to test if development of hyperalgesia during acute (ASD) and chronic (CSD) sleep deprivation correlates with the cumulative relative sleep loss. Left, accumulation of relative sleep loss over four consecutive days of daily 6 h sleep deprivation. Dashed line represents cumulative wake in undisturbed sleep and black line represents cumulative wake during daily 6 h sleep deprivation. Stars represent time of sensory testing. Note: the amount of relative sleep loss is the same as relative wake gain. Right, lack of correlation between percentage of hyperalgesia and relative sleep deficit during acute and chronic sleep deprivation. **(C)** “Extended wake” model to test if development of hyperalgesia during acute (ASD) and chronic (CSD) sleep deprivation correlates with the amount of extended wake coupled with carryover sleep debt. Left, quantification of the sleep debt (in blue) that builds up during chronic sleep deprivation protocol (in red). Stars represent time of sensory testing. Right, the development of heat hyperalgesia during acute and chronic sleep deprivation correlates with the amount of extended wake coupled with the carryover sleep debt. Goodness to fit: R squared = 0.9916.

We quantified the cumulative wake amount over 5 consecutive days of experimental sleep restriction (6 h of sleep deprivation daily, starting at light onset) in mice and confirmed that despite a normal homeostatic sleep response (increased in NREMS SWA and NREMS amount) occurring each day during the sleep opportunity period, there was an accumulation of sleep loss/deficit over time (Alexandre et al., 2017). We compared the degree of heat hyperalgesia caused by an acute, extended wakefulness of 6, 9, and 12 h with the effects of the repeated sleep restriction protocol (6 h of extended wake per day) and found they both gradually increased, with a plateau after 12 h acute sleep deprivation or 4 d of sleep restriction (Figure 6A). Interestingly, we found that the development of heat hyperalgesia fits best when incorporating the carryover amount of wake (i.e., the sleep debt at the beginning of the active period, in blue) with the extended wake (in red, Figure 6B), rather than the relative cumulative sleep loss measured at the time of testing (i.e., at the end of the sleep deprivation session; in pink; Figure 6C).

Overall, the cumulative effects of sleep debt on pain could explain why heat and mechanical pain hypersensitivity takes several days to develop in complex chronic settings that are associated with a moderate total sleep loss in humans. For example, the forced awakenings protocol, which causes a 4 h total sleep loss per day, is insufficient to increase heat or mechanical (pinprick) pain after a single night (see Figure 5), but increases pain responses to both modalities after two consecutive nights (Smith et al., 2007; Iacovides et al., 2017; Irwin et al., 2022). As the individual has to maintain wakefulness throughout the day despite the homeostatic sleep pressure caused by the first night of forced awakenings (4 h sleep loss and broken sleep continuity), this maintained wakefulness could represent an additional extended wake period. After the second night of forced awakenings, the net result is accumulation of sleep debt coupled with an episode of extended wakefulness (similar to an acute night of delayed sleep onset), which causes heat and mechanical hypersensitivity. Similarly, platform-over-the-water protocols occur over several days, causing a buildup of sleep debt that increases heat pain hypersensitivity over time. Finally, these dynamic processes could explain how longitudinal protocols where individuals go through stage-specific sleep deprivations followed by total sleep deprivation can lead to complex and sometimes misleading interpretations.

In conclusion, both clinical and preclinical studies confirm a strong effect of sleep disturbances on pain sensitivity in a highly conserved manner. However, different sleep disturbances affect preferentially specific components of the pain response. REMS deprivation is not associated with a strong increase in pain responses, and the major driver for hyperalgesia appears to be the total amount of sleep lost. Further, the dynamics of sleep loss reveal potential mechanistic insights: sleep loss caused by extended wake is strongly associated with heat and mechanical pain hypersensitivity; whereas a similar loss achieved by disrupting sleep continuity causes a loss of diffuse noxious inhibitory controls. Finally, the sleep debt and its deleterious effects on pain sensitivity are cumulative over days, despite a preserved homeostatic sleep rebound, which over time could contribute to the complex pain responses profile observed in chronic sleep disturbances settings.

## Part 5: Possible sites for pain modulation by sleep loss

Nociceptive signals can be amplified at many levels along the pain pathways (see Figure 1A). Below we describe where the nociceptive input might be enhanced by acute and chronic sleep loss, and the potential mechanisms.

### Nociceptors

Nociceptors can lower their activation threshold, notably when exposed to pro-inflammatory cytokines/chemokines, trophic factors or even foreign bodies (Chiu et al., 2013), a process known as peripheral sensitization. A major source of proinflammatory cytokines are immune cells. Immunity is composed of the innate and the adaptive immune systems and both are heavily regulated by circadian and sleep-wake cycles (Imeri and Opp, 2009; Lange et al., 2010; Besedovsky et al., 2012; Dimitrov et al., 2015; Irwin and Opp, 2017; Liu et al., 2021) and strongly affected by sleep loss (Bryant et al., 2004). An acute total sleep loss of 3–4 h is sufficient to alter the innate immune system that consists of monocytes (blood-circulating precursors of macrophages), macrophages, eosinophils, neutrophils, basophils, mast cells, and natural killer cells (NK cells). In particular, the number of circulating neutrophils and monocytes increase after sleep loss in humans (Irwin et al., 2008; Christoffersson et al., 2014; Faraut et al., 2015; Lasselin et al., 2015), rats (Ibarra-Coronado et al., 2015) and mice (Guariniello et al., 2012). In these cells, sleep loss enhances the transcriptional activity of the proinflammatory nuclear factor NFkB, which in turn promotes the production and release of TNFα, IL-1β, IL-6, IL-8, and C-reactive protein (Hu et al., 2003; Haack et al., 2007; Irwin et al., 2008, 2015, 2022; Yehuda et al., 2009; Aho et al., 2013; Carroll et al., 2015; Dimitrov et al., 2015). These cytokines can bind to and sensitize nociceptors directly (Brenn et al., 2007; Binshtok et al., 2008; Kawasaki et al., 2008; Stemkowski et al., 2015). In addition, IL-1β triggers the induction of cyclooxygenase 2 (COX2) in macrophages (Samad et al., 2001) which leads to the production of the prostaglandin PGE2, a potent sensitizer of nociceptors (Chapman and Dickenson, 1992; Chen et al., 1999). PGE2 levels correlate with migraine and headache complaints after sleep deprivation (Haack et al., 2007, 2009). The overall shift into a pro-inflammatory profile while factors that can terminate the immune response (i.e., anti-inflammatory cytokines and chemokines), could extend the duration of inflammation.

T cells and B cells (adaptive immune system) are further classified into helper (Th), regulator (Treg), killer (cytotoxic) and memory T cells, and they produce a highly targeted cellular response that relies on the prior exposure to the foreign bodies. Sleep loss durations lasting from 3 to 21 days affect the adaptive immune system, with an overall downregulation of the “cellular immune response” carried out by Th-1 lymphocytes in favor of Th-2 and Th-17 lymphocytes-mediated responses in humans and rodents (Van Leeuwen et al., 2009; Axelsson et al., 2013; Nunes et al., 2018). This promotion of Th-2-mediated responses could be further strengthened by PGE2 produced by monocytes/macrophages (Harris et al., 2002). IL-17 directly causes pain (Pinto et al., 2010) by sensitizing nociceptors (Segond von Banchet et al., 2013), while the pro-nociceptive effects of IL-22 are possibly indirectly mediated by neutrophil recruitment (Pinto et al., 2015). IL-22 can also promote the differentiation into Th-2 lymphocyte profile, further amplifying this shift (Lou et al., 2017). Finally, the dysregulation of B cell function after chronic sleep loss impairs the efficiency of immunization (Zielinski and Krueger, 2011; Prather et al., 2012).

**Figure 7:**
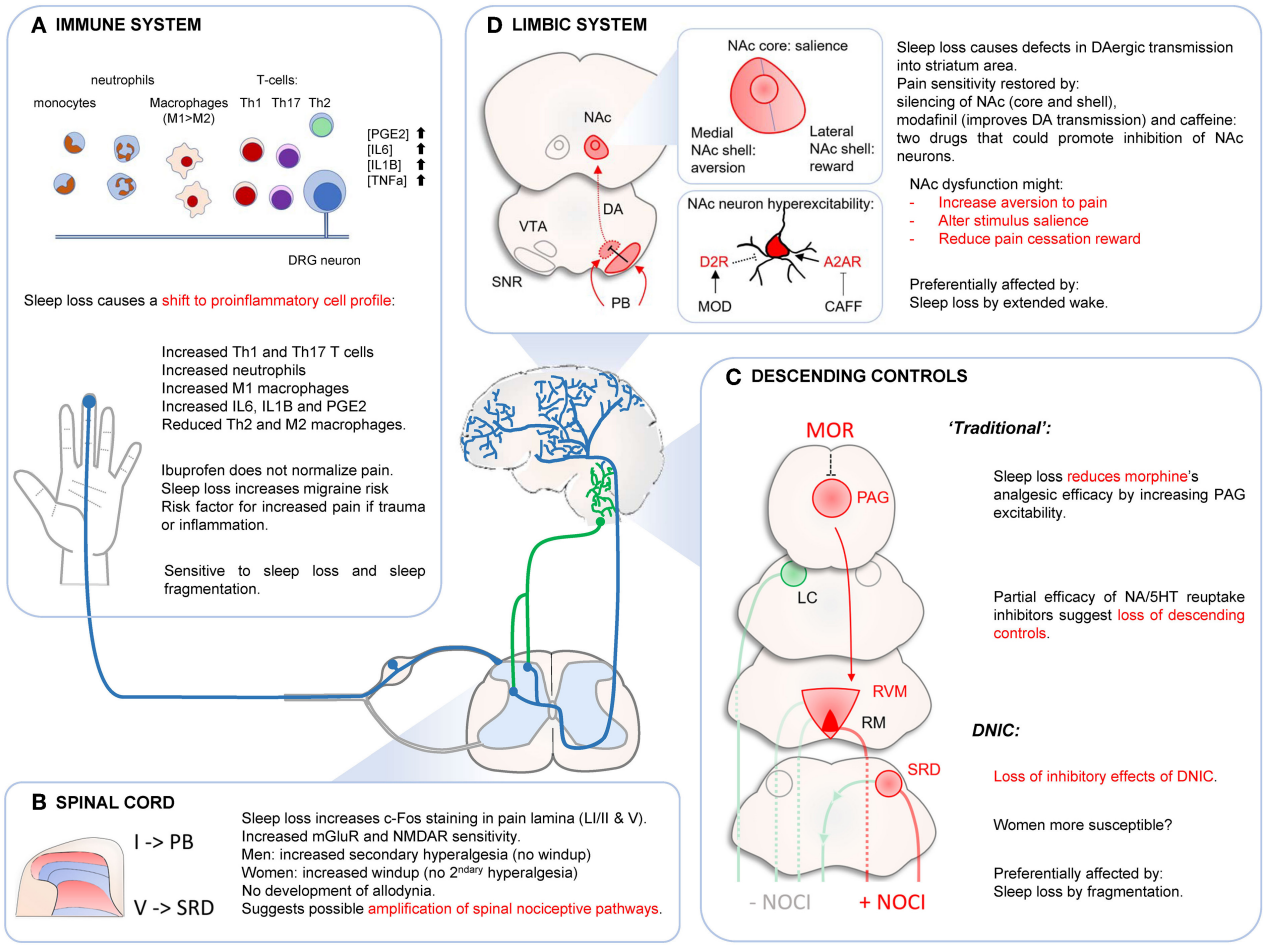
Effects of sleep loss on immune system **(A)**, spinal cord **(B)**, descending controls **(C)** and limbic system **(D)**. **(A)** Sleep disturbances cause a shift toward pro-inflammatory responses. This could promote risk of migraine attacks and maladaptive immune response in case of injury or infection. **(B)** Sleep loss might increase responsiveness of spinal nociceptive circuits, without causing central sensitization. **(C)** Sleep disturbances decrease morphine's analgesic effects probably at the PAG level. This dysregulation of PAG neurons might affect “traditional” descending controls. DNIC are strongly affected by sleep loss by fragmentation. This effect seems stronger in women. **(D)** Sleep loss alters normal NAc function, which could affect several components of the pain response such as aversion to pain, determination of stimulus salience or pain cessation reward. Sleep loss causes hyperexcitability of NAc neurons and reducing this (local silencing, increase D2R signaling, blocking A2AR signaling) restores pain sensitivity. DAergic transmission might also be impaired by abnormal VTA neurons inhibition by SNR GABAergic neurons contacted by PB neurons. Dysfunctions of NAc circuits might mediate evoked pain hypersensitivity after sleep loss, especially after extended wake periods. NAC, nucleus accumbens; VTA, ventral tegmental area; SNR, substantia nigra pars reticulate; PB, parabrachial nucleus; PAG, periaqueductal gray area; LC, locus coeruleus; RVM, reticular ventromedial area; RM, Raphe Magnus; SRD, subnucleus reticularis dorsalis.

Overall, these results indicate that sleep loss initially promotes the proinflammatory component of the innate immune response (monocytes/macrophages, neutrophils) and then a shift toward allergic reactions and autoimmunity at the adaptive immune system level (Th2/Th17). Because these changes are associated with an increase in pronociceptive signaling (IL-6, IL-1β, TNFα) and a reduction of antinociception (IL2), they could contribute to pain hypersensitivity or a delayed recovery after tissue injury. However, administration of the non-specific COX1/2 inhibitor ibuprofen does not reduce acute or chronic sleep loss-induced heat or mechanical evoked pain hypersensitivity (Wodarski et al., 2015; Alexandre et al., 2017), suggesting a limited role of inflammatory processes in these symptoms. In contrast, spontaneous pain complaints or migraine attacks, whose frequency increases after repeated nights of insufficient sleep (Haack and Mullington, 2005; Haack et al., 2007; Odegard et al., 2011) might be mediated by the sleep loss-induced increase in prostaglandin levels (Haack et al., 2009). Finally, sleep loss-induced increase in IL-6 or TNFα levels might aggravate the defects in emotional status (Hunt et al., 2021), possibly by crossing the blood brain barrier and reaching the mesolimbic system (Hunt et al., 2022) (Figure 7A).

### Spinal cord

The spinal cord is a major site for nociceptive signal modulation. Only a small fraction of spinal neurons project to brain structures and they are mostly located in the lamina I and V of the dorsal horns (Todd, 2010). These neurons are under strong modulatory controls, both excitatory and inhibitory that originate both locally (“segmental controls”) and at the supra-spinal level (mostly located within the rostroventral medulla and the pons); (Alexandre et al., 2020). Changes in the excitatory/inhibitory balance in the dorsal horns of the spinal cord will affect the nociceptive signal sent to the brain. One potent form of spinal plasticity is called central sensitization, which corresponds to a heightened functional state of the spinal nociceptive neurons where their activation threshold is reduced, they produce stronger and longer responses upon activation by noxious stimuli (a symptom referred to as hyperalgesia), and they can be activated by innocuous stimulations (allodynia) (Latremoliere and Woolf, 2009). Sleep loss could enhance spinal excitability by modulating both segmental and supraspinal controls.

Proinflammatory cytokines and PGE2 produced at the periphery after sleep loss can penetrate the spinal cord and promote nociceptive transmission (Baba et al., 2001; Samad et al., 2001; Latremoliere and Woolf, 2009). In addition, sleep loss increases sensitivity of NMDAR and mGluR in the dorsal horns (Wei et al., 2007, 2010), and this is associated with an increase in mechanical pain in rats. Capsaicin (active ingredient of red-hot chili peppers that activate TRPV1 channels) can be used to trigger central sensitization and measure associated behavioral changes caused by spinal plasticity. Four nights of platform over water paradigm (rats) increased c-fos immunoreactivity in the trigeminal nucleus upon supradural capsaicin infusion, suggesting increased spinal neuronal excitability, or longer time to return to normal sensitivity at the first relay of nociceptive transmission (Kim et al., 2019). After two nights of forced awakenings (humans), women displayed increased windup pain (but not men), while men developed mechanical secondary hyperalgesia (but not women) (Smith et al., 2019). These results indicate a possible spinal amplification of nociceptive signaling with probable sex difference. However, the lack of development of secondary allodynia in sleep-deprived subjects suggests that forced awakenings and moderate sleep loss do not trigger a state of central sensitization (where innocuous stimuli activate spinal nociceptive neurons and cause pain), but rather specifically amplify the nociceptive/pain signal. This amplification could involve a spinal facilitation, suggested by the increase in c-fos spinal staining and windup pain readout (Figure 7B). The increased pain ratings during repeated nociceptive stimulations could also involve supraspinal changes (in brainstem, thalamus, cortex) or an increasing discomfort or anxiety with repeated painful stimulations.

Several lines of evidence suggest sleep loss could also dysregulate descending controls into the spinal cord. Traditional descending inhibitory controls are mostly recruited by the spino-periaqueductal gray (PAG) pathway, which originates from lamina I spinal projection neurons (Gauriau and Bernard, 2002). The PAG is critical for the analgesic effects of morphine (Fields, 2004; Ossipov et al., 2014). Under normal conditions, morphine binds to inhibitory PAG interneurons, which release their brake on glutamatergic PAG neurons that project onto the pain-inhibiting RVM neurons (“OFF-cells”). This disinhibition of RVM “OFF cells” allows the recruitment of inhibitory descending controls. After a single night of sleep deprivation, there is a loss of morphine analgesic efficacity in humans and rodents (Ukponmwan et al., 1984; Nascimento et al., 2007; Steinmiller et al., 2010; Skinner et al., 2011; Alexandre et al., 2017), which could be due in part to an hyperexcitability of GABAergic PAG interneurons preventing the recruitment of inhibitory descending pathways from the RVM (Tomim et al., 2016).

Administration of amitriptyline, a non-selective 5-HT/NA reuptake inhibitor (which increases levels of these monoamines in the spinal cord), improves heat pain hypersensitivity in rats after sleep loss (but not mechanical pain hypersensitivity), thus supporting the hypothesis of a partial loss of noradrenergic and serotoninergic descending controls (Wei et al., 2008; Wodarski et al., 2015). Amitriptyline has also been shown to be an agonist of Kv7.2/7.3 channels (Punke and Friederich, 2007), and selective activation of these channels phenocopied amitriptyline in sleep-deprived animals (Wodarski et al., 2015), which could suggest Kv7.2/7.3 are involved in sleep loss-induced heat hyperalgesia. However, one caveat is that activation of Kv7.2/7.3 channels causes analgesia in well-rested animals (Teng et al., 2016) and are not restricted to the RVM-spinal projections. Finally, in parallel to this possible loss of inhibitory descending controls, sleep loss increases RVM cholecystokininergic transmission, which promotes pro-nociceptive descending controls (“ON-cells”; Tomim et al., 2016).

Overall, several studies indicate a probable change in spinal-midbrain pathways' excitability after sleep loss. Sleep disturbances do not trigger a frank state of central sensitization, but rather a relative gain in pronociceptive signaling, in part mediated by changes in RVM spinal projections (Figure 7C). Whether this plasticity also involves intrinsic changes within the spinal nociceptive circuits remains unclear.

### Brain: Mesolimbic system

While the spino-thalamo-cortical pathway plays a key role for the sensory-discriminative component of pain and the spino-parabrachial pathway generates its emotional (escape and aversive memory) and autonomic (heart rate, pupil dilatation, sweating) components (Gauriau and Bernard, 2002; Tracey et al., 2019; Chiang et al., 2020), several brain areas associated with cognitive and emotional processes can also modulate the final pain response based on its context (e.g., attention to pain, distraction analgesia, pain tolerance, etc.). The mesolimbic system could play a major role in this contextualization of the pain sensation, notably by determining the salience of the stimulus (Knutson et al., 2001; Roitman et al., 2005; Cooper and Knutson, 2008; Al-Hasani et al., 2015) and by modulating the reward system during and after a pain stimulation (Lammel et al., 2014; Navratilova et al., 2015; Yang et al., 2021). Several lines of evidence have recently pointed to a critical role of the mesolimbic system, notably the nucleus accumbens (NAc) on the proalgesic effects of sleep loss on pain (Finan et al., 2013; Sardi et al., 2018b; Alexandre et al., 2020). The NAc is subdivided in three functionally distinct areas: the NAc core, the medial part of the NAc shell, and the lateral part of the NAc shell (Lammel et al., 2014), which all likely play a distinct role in the overall pain experience. The NAc core is critical to determine the salience of stimuli (Chen and Bruchas, 2021), the medial NAc shell is involved in the aversive-encoding prediction of an unpleasant stimulus (including pain) (de Jong et al., 2019); the lateral NAc shell is a key structure for the reward system (Lammel et al., 2014; de Jong et al., 2019). NAc activity is strongly modulated by dopamine released from the ventral tegmental area (VTA) *via* mostly segregated pathways: the medial VTA projects to the NAc core and the medial NAc shell, while the lateral VTA almost exclusively projects onto the lateral NAc shell (Lammel et al., 2014). A painful stimulation causes an activation of DA neurons located in the most ventral part of the medial VTA (Brischoux et al., 2009; Lammel et al., 2014; de Jong et al., 2019), presumably reinforcing aversion (de Jong et al., 2019), while it mostly inhibits dopamine release in the lateral NAc shell (de Jong et al., 2019), likely blocking pleasant/reward pathways during ongoing pain. This inhibition of lateral VTA neurons is notably mediated by GABAergic SNR neurons, which are directly contacted by lateral parabrachial nucleus neurons (Yang et al., 2021). At the cessation of the pain stimulation, there is a rebound of dopamine release onto neurons of the lateral NAc shell, a phenomenon known as “pain cessation reward” (Navratilova et al., 2015).

Overall, these studies indicate that the dynamics of dopamine release onto different regions of NAc are extremely important for an adequate pain response. Functional magnetic resonance imaging (fMRI) studies found that sleep loss strongly alters these activation dynamics of NAc upon noxious stimulation (Krause et al., 2019; Seminowicz et al., 2019), notably through a defect in D2R/D3R transmission (Volkow et al., 2008, 2012). In rodents, excitotoxic lesions, local silencing of the NAc (Sardi et al., 2018a,b) or administration of modafinil (Alexandre et al., 2017), which enhances DAergic tone (Boutrel and Koob, 2004), normalize pain sensitivity without any analgesic activity on their own. This dysregulation of VTA-NAc function upon painful stimulation after sleep loss could affect how a nociceptive stimulus is evaluated (salience), how pain (or its cessation) is going to be anticipated, or dampen the positive sensation associated with the termination of a painful stimulus. VTA neurons are strongly implicated in sleep-wake behaviors and both DAergic and GABAergic neurons of the VTA are active during wakefulness and quiet during NREMS (Eban-Rothschild et al., 2016). Their sustained activation during extended wake coupled with a limited recovery during shorter sleep opportunity could contribute to the accumulation of sleep loss-induced hyperalgesia. In addition, an increased input from the spinal-parabrachial pathway after sleep loss (originating from lamina I nociceptive neurons) could in turn overactivate SNR GABAergic neurons projecting to the lateral VTA, and thereby contribute to the reduced DAergic transmission onto NAc neurons and disrupt normal activation dynamics of this nucleus upon painful stimulation (Figure 7D).

The majority of NAc neurons expressing D2R express the excitatory adenosine A2A receptors (Valjent et al., 2009) (A2AR). Blockade of A2AR, notably by caffeine, normalizes pain in sleep-deprived animals (Alexandre et al., 2017; Sardi et al., 2018a) while local administration of A2AR agonists extends sleep loss-induced hyperalgesia in rats (Sardi et al., 2018b). Adenosine accumulates with wakefulness, which would contribute to the overexcitability of NAc neurons during extended wake.

Altogether, these data suggest that sleep loss might lead to a state of hyperexcitability of D2R/A2AR NAc neurons, which would be caused by a combination of reduced function of D2R (internalization, or decoupling of G-protein), but also an accumulation of adenosine during wakefulness activating A2AR (Gq-coupled). Restoring an inhibitory brake on these neurons (by promoting D2R or blocking A2AR) could restore normal pain sensitivity by maintaining the normal excitatory/inhibitory balance required for the different subnuclei of the NAc to function (Figure 7D). Finally, it is worth mentioning that in humans, laser-evoked potential signal was reduced after sleep loss, despite an increase in pain ratings (Tiede et al., 2010; Azevedo et al., 2011), which would suggest that the nociceptive input might not be increased in higher brain structures, but rather by promoting how the signal is perceived and contextualized—two major features of the mesolimbic system.

## Conclusions

Despite significant differences in sleep-wake patterns between humans and rodents and a great heterogeneity in protocols used to disturb sleep, the effects of sleep disturbances on pain sensitivity are remarkably conserved across species. In addition, in all species assessed, females developed more pain hypersensitivity than males, and this might involve distinct neural mechanisms. We propose that the major driver for pain hypersensitivity is the total amount of sleep lost, while REMS loss by itself does not seem to have a direct effect on pain. REMS deprivation however increases the febrile response and might contribute to impaired recovery upon injury (Sutton and Opp, 2014; Wang et al., 2015; Vanini, 2016; Dai et al., 2022). The dynamics of sleep loss might affect different components of the pain response: sleep loss caused by extended wakefulness preferentially increases pain perception, whereas interrupted and limited sleep strongly dysregulates descending controls such as DNIC, especially in women.

Sleep disturbances affect pain pathways at several anatomical levels. Insufficient sleep causes a shift in the immune system toward a proinflammatory profile. While the increase of these proinflammatory factors (notably prostaglandins and cytokines) at the periphery is not sufficient to trigger evoked pain hypersensitivity, these agents might promote migraine as well as the risk of developing abnormal pain after injury (and inflammation). Sleep disturbances might enhance pain transmission at the spinal level, without however causing a state of full central sensitization (i.e., no development of allodynia). This increased spinal excitability has a strong sexual dimorphism, which could be caused by sex-dependent spinal mechanisms or changes from supraspinal structures. For example, sleep disturbances affect DNICs only in women, while men are mostly spared. Sleep loss causes an overexcitability of PAG neurons, which reduces morphine analgesic efficacy and could also alter “traditional” descending controls. Finally, sleep loss caused by an extended wake, similar to that experienced by a large body of the general population, might preferentially affect the mesolimbic system. This system plays a key role in the emotional and cognitive component of the pain response. Sleep loss-induced dysfunctions of the mesolimbic system could increase pain by promoting the attention to pain, amplifying catastrophizing (expectation of when a painful stimulus will stop), and reducing distraction analgesia.

Because the deleterious effects of sleep loss on pain sensitivity are cumulative, they likely contribute to the complex sleep and pain profiles observed in chronic clinical settings. Understanding how specific pain components are dysregulated by different sleep disturbances will help decipher how they interact over time, and the best strategies to promote sleep features that normalize pain sensitivity.

### Methodology

We searched for articles studying the effects of sleep disturbances on pain on PubMed and Google Scholar. Key words included “sleep deprivation”; “sleep loss”; “chronic sleep deprivation”; “chronic sleep loss”; “chronic sleep restriction”; “REMS deprivation”; “total sleep deprivation”; “pain”; “nociception” and their combination. For this review, we selected studies that measured evoked pain sensitivity in laboratory settings (no diaries). Only experiments in healthy subjects were used. For animal studies, we only used data from healthy animals (in some cases, non-injured animals that acted as controls to injury models). Determination of pain hypersensitivity (or normal pain sensitivity) was analyzed for all time points reported within the studies. In studies using multiple sleep disruption protocols, we only analyzed the pain results from the first disruption used. If studies not obtained by the PubMed or Google Scholar searches were identified by reading manuscripts, they were added to this study.

## Data availability statement

The original contributions presented in the study are included in the article/supplementary material, further inquiries can be directed to the corresponding authors.

## Author contributions

KK, CA, and AL analyzed the data and wrote the manuscript. All authors contributed to the article and approved the submitted version.
